# Case Report: Synchronous cutaneous and pineal metastases from gastric adenocarcinoma—inter-lesion biomarker heterogeneity and multi-line targeted therapy

**DOI:** 10.3389/fonc.2026.1924850

**Published:** 2026-08-12

**Authors:** Qing Shen, Wanqian Zhang, Ying Chen

**Affiliations:** 1 Department of Medical Oncology, The First Hospital of China Medical University, Shenyang, China; 2 Key Laboratory of Anticancer Drugs and Biotherapy of Liaoning Province, The First Hospital of China Medical University, Shenyang, China; 3 Liaoning Province Clinical Research Center for Cancer, The First Hospital of China Medical University, Shenyang, China; 4 Key Laboratory of Precision Diagnosis and Treatment of Gastrointestinal Tumors, Ministry of Education, The First Hospital of China Medical University, Shenyang, China

**Keywords:** cutaneous metastases, gastric adenocarcinoma, gastric cancer, heterogeneity, pineal metastases

## Abstract

Gastric adenocarcinoma simultaneously metastasizing to the skin and pineal gland represents an extraordinarily rare clinical manifestation. Pineal metastases account for less than 1% of all adult intracranial tumors and seldom originate from gastrointestinal primary lesions, and this atypical metastatic combination frequently results in delayed diagnosis and complicated clinical decision-making. Distinct molecular profiles between primary and metastatic lesions create additional obstacles to personalized treatment, and relevant standardized management strategies for such rare cases have not been well established. We describe a 43-year-old woman with advanced gastric adenocarcinoma complicated by synchronous cutaneous and pineal metastases. Pathological evaluation confirmed the gastric origin of the cutaneous lesions, while the pineal lesion was presumptively classified​ as metastatic based on molecular concordance and clinical context. Guided by variable biomarker status across metastatic sites, sequential individualized targeted regimens were delivered, yielding sustained disease stabilization and satisfactory long-term survival. This case supplements clinical data on rare distant metastatic patterns of gastric cancer and highlights that repeated multi-site genetic testing is essential to formulate precise therapeutic plans for heterogeneous advanced gastrointestinal malignancies.

## Introduction

Gastric adenocarcinoma is a malignant epithelial neoplasm with a strong propensity for metastasis. However, metastasis to the skin and the pineal gland is exceptionally rare, making such cases clinically significant. The incidence of cutaneous metastasis from gastric adenocarcinoma ranges from 0.2% to 2.2%. These cutaneous metastases (CMs) typically present as nodules, erythema, or indurated plaques (1–4).

Pineal gland metastasis is even rarer, with only sporadic case reports documented in the literature (5). In the reported case, the simultaneous presence of both skin and pineal gland metastases was observed in the same patient, with the metastatic lesions exhibiting distinct genetic profiles compared to the primary tumor. This extraordinary clinical scenario challenges our understanding of metastatic pathways and tumor heterogeneity. The case report describes​ a presumptive case of synchronous cutaneous and pineal metastases from gastric adenocarcinoma, highlighting​ the challenges of diagnosing anatomically inaccessible lesions and the potential utility of liquid biopsy in guiding individualized therapeutic decisions.

## Case description

### Diagnostic assessment

In May 2020, a 43-year-old female patient was diagnosed with gastric adenocarcinoma complicated by ovarian metastasis. In September 2020, following multidisciplinary team (MDT) deliberation and thorough shared decision-making with the patient, she underwent distal gastrectomy and bilateral oophorectomy. According to the pathological Tumor-Node-Metastasis (pTNM) classification, the postoperative stage was IV (T4aN3bM1), with cancer cells identified in the peritoneal lavage fluid. Immunohistochemical staining demonstrated a proficient mismatch repair (pMMR) status, and the human epidermal growth factor receptor 2 (HER2) was negative. Following surgery, the patient developed sequential cutaneous metastases: a left chest wall erythematous lesion was pathologically confirmed as metastatic gastric adenocarcinoma in May 2022 (**Supplementary Figure 1**), and a new right chest wall metastasis was identified in April 2023. In August 2023, she presented with progressive neurological symptoms highly suggestive of central nervous system involvement, including bilateral temporal dull pain, persistent blurred vision (uncorrected visual acuity 0.3 in both eyes, down from 1.0 six months prior), and mild gait instability. Cranial CT on August 23 revealed a 1.0×0.6 cm nodule in the suprasellar cistern with obvious enhancement. Subsequent multi-sequence, multi-planar MRI revealed an 8 mm enhanced isointense nodule in both the suprasellar cistern and pineal gland region, highly suggestive of metastatic tumors (**Supplementary Figure 2**). The lesion exhibited isointensity on T1-weighted imaging and marked homogeneous enhancement post-contrast, accompanied by multifocal enhancing lesions in the suprasellar cistern without evidence of calcification or cystic components. Concurrent endocrine abnormalities were detected, including adrenal cortical insufficiency, hypothyroidism, and polyuria (**Supplementary Tables 1**–**3**). Dynamic monitoring showed markedly reduced plasma cortisol (8:00) at 41.58 nmol/L, serum free thyroxine (FT4) at 7.43 pmol/L, free triiodothyronine (FT3) at 2.32 pmol/L, and a 24-hour urine volume ranging from 6000 to 8000 mL. Molecular profiling revealed significant inter-lesion heterogeneity in HER2 and combined positive score (CPS) status across metastatic sites (**Table 1**). Molecular profiling was performed using the GeneSeeq SHIELDING™ circulating tumor DNA (ctDNA) panel (425-gene) by SHINYGENE Molecular Diagnostics Co., Ltd. This next-generation sequencing-based assay covered core cancer-related genes (including B-Raf proto-oncogene, serine/threonine kinase—BRAF​ and HER2) and was conducted according to the manufacturer’s protocols and laboratory standard operating procedures (SOPs).

**Table 1:** Biomarker status at different tumor sites.

Site	HER2 Status	CPS	BRAF V600E
Stomach	Negative	1	Not assessed
Liver	Unknown	Unknown	Unknown
Abdominal Cavity	Unknown	Unknown	Unknown
Left Chest Wall	2+ (FISH non-amplified)	<1	Not assessed
Right Chest Wall	2+ (FISH non-amplified)	3	Not assessed
Anastomosis	Negative	1	Not assessed
CSF ctDNA	Not assessed	Not assessed	Positive (VAF 47.67%)

CPS, Combined Positive Score; CSF, cerebrospinal fluid; VAF, variant allele frequency; FISH, fluorescence *in situ* hybridization.

“Unknown” indicates no tissue was available for testing. “Not assessed” indicates specimens were available but the specific biomarker was not evaluated (HER2/CPS require tissue-based immunohistochemistry, not applicable to CSF; BRAF testing was not performed on archival tissue specimens). Molecular data for the pineal lesion are limited to CSF ctDNA findings, as tissue acquisition was precluded by high procedural risk.

### Therapeutic intervention

Targeted hormone replacement therapy was immediately initiated to manage endocrine dysfunction. Intravenous hydrocortisone was administered and tapered to oral maintenance, levothyroxine was prescribed for hypothyroidism, and one-eighth (⅛) of a tablet of desmopressin was administered for polyuria. This intervention led to significant improvement, stabilizing urine volume at 2000–3000 mL and normalizing FT4/FT3 levels without interrupting subsequent anti-tumor therapy. Given the patient’s refractory disease and molecular profile, a personalized systemic therapeutic strategy was adopted sequentially (**Table 2**). Due to a history of Sjögren’s syndrome, immune checkpoint inhibitors (ICIs) were excluded from consideration. For the fourth-line treatment, dabrafenib plus trametinib was initiated targeting the BRAF V600E mutation detected in the peripheral blood ctDNA. Subsequently, given the emergence of HER2-low subclones​ (IHC 2+/fluorescence *in situ* hybridization (FISH) non-amplified) in peripheral metastases, the fifth-line regimen was shifted to Disitamab vedotin combined with apatinib and S-1. Additionally, CyberKnife radiotherapy was administered to the pineal lesion from September 19 to 25, 2023, with gross tumor volumes receiving 25 Gy and 35 Gy in 5 fractions, respectively.

**Table 2:** Detailed treatment timeline and clinical outcomes.

Treatment line	First-line	Second-line	Third-line	Fourth-line	Fifth-line	Sixth-line	Seventh-line	Outcome
Time Point	2020-11-02	2020-12-25	2022-05-13	2022-07-08	2022-08-24	2023-04-25	2023-09-09	2023-11-30
Lesion Site	Abdominal cavity	Abdominal cavity, Liver	Left chest wall	Left chest wall, Anastomosis	Left chest wall, Anastomosis	Right chest wall (newly occurred), Anastomosis	Brain, Lung	
Treatment Regimen	Sox	Nab-paclitaxel + Apatinib	Irinotecan + Apatinib	Dabrafenib + Trametinib	Disitamab Vedotin + Apatinib + S-1	Disitamab Vedotin + TAS-102 + Apatinib	Dabrafenib + Cetuximab + Anlotinib +CyberKnife for brain	
Clinical Response	PD	CR	PD	PD	PR	SD	PD	Death
TTP (months)	1.7m	16.6m	1.8m	1.5m	8m	4.5m	2.7m	

TTP, Time To Progression; PD, Progressive Disease; CR, Complete Response; PR, Partial Response; SD, Stable Disease; SOX, S-1 plus oxaliplatin; CSF, cerebrospinal fluid; VAF, variant allele frequency.

Cutaneous metastases:​ Left thoracic wall (biopsied May 13, 2022); right thoracic wall (biopsied April 25, 2023).

Molecular rationale:​ Fourth-line dabrafenib/trametinib was initiated July 8, 2022, guided by rising ctDNA BRAF V600E VAF (0.5% in June 2021 → 13.2% in July 2022). At CNS progression, blood VAF was 25.66% and CSF VAF was 47.67%.

Limitations:​ BRAF testing was not performed on primary/extracranial lesions; blood-CSF concordance supports a clonal association with the pineal lesion.

Seventh-line therapy:​ Included dabrafenib plus cetuximab and anlotinib, combined with CyberKnife radiosurgery for the pineal lesion.

### Follow-up and outcomes

The patient achieved a partial response to the Disitamab vedotin-based regimen lasting 8 months. Following sixth-line therapy with Disitamab vedotin and TAS-102, the disease remained stable for 4.5 months. Throughout the treatment course, the patient maintained a satisfactory quality of life despite multiple lines of therapy. Notably, the endocrine abnormalities coincident with pineal metastasis were well-controlled with hormone replacement. The patient’s overall survival from the initial diagnosis reached 42.9 months.

## Discussion

Synchronous cutaneous and pineal metastases from gastric adenocarcinoma are extremely rare. To our knowledge, this represents the first reported case presumptively classified​ as such, based on molecular concordance and clinical context. Although histopathological confirmation of the pineal lesion was not possible due to its location, the case highlights the potential of integrating liquid biopsy with radiological and clinical data to characterize inaccessible metastatic sites.

**Figure 1:**
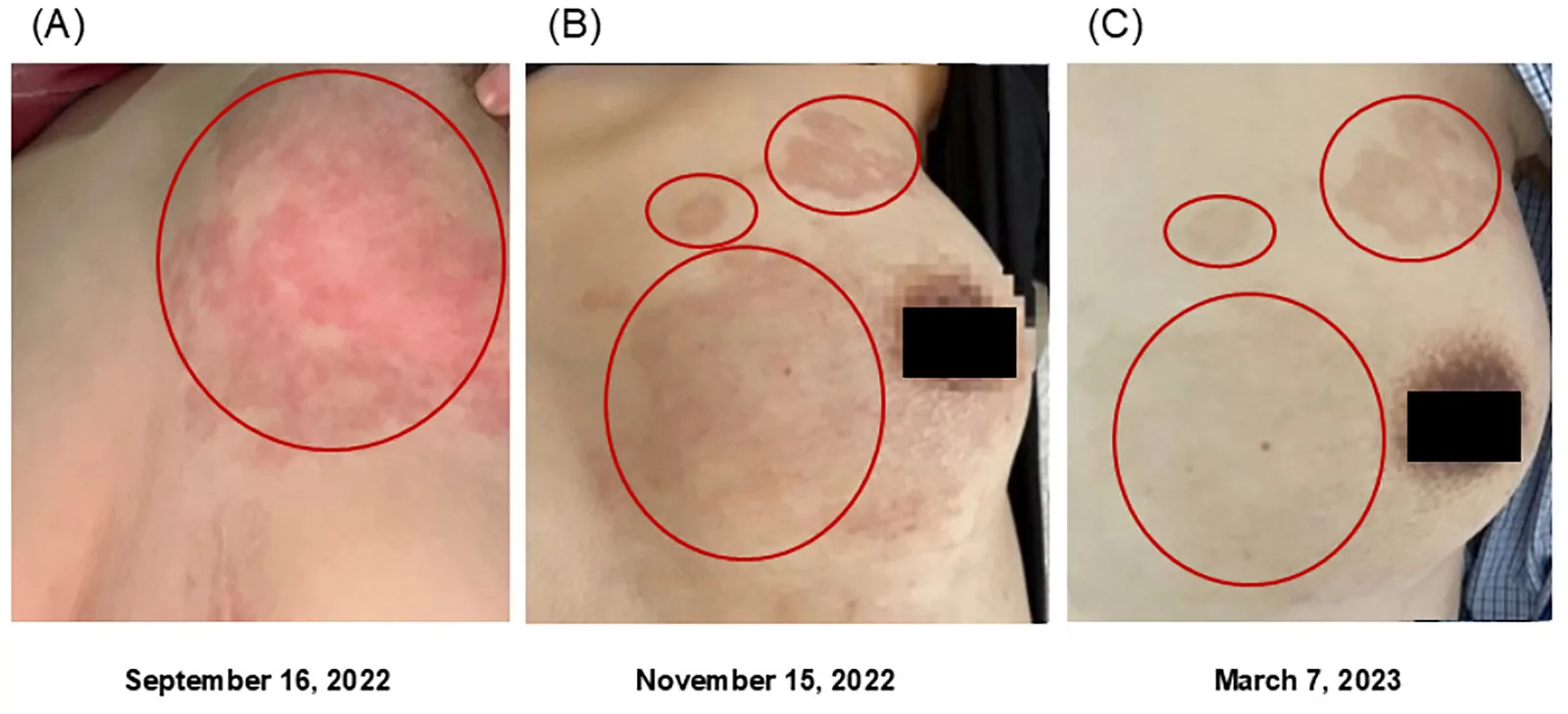
Serial clinical photographs of the left chest wall erythematous nodular lesions showing progression and subsequent regression. **(A)** Acute presentation on September 16, 2022, with prominent erythema and nodularity; **(B)** Partial regression on November 15, 2022, with reduced erythema and size; **(C)** Near-complete resolution on March 7, 2023, with only residual hyperpigmentation. Red circles indicate the primary lesion area.

Gastric adenocarcinoma is a major global health issue, often diagnosed late. Its metastatic spread is complex and heterogeneous, commonly involving the peritoneum, liver, lung, and bone (6–8). CMs from gastric adenocarcinoma are rare, with histopathology showing adenocarcinoma cells and immunohistochemical markers (CK7, CK20, CDX2) confirming gastric origin (9). While pathological diagnosis is the gold standard, other tools like F-18 FDG PET-CT and dermoscopy are also important (10, 11). CMs often appear as erythematous or violaceous nodules, sometimes mimicking erysipelas (12), and may precede the primary tumor diagnosis (13). In this case, a left chest wall rash was confirmed as metastatic gastric adenocarcinoma (**Figure 1**), with a later contralateral lesion indicating progression. Systemic therapy is standard (14, 15), but some patients with localized disease may benefit from surgery (16). Although the patient had a well-documented history of Sjögren’s syndrome that had been stably controlled over the long term, we elected not to pursue ICIs therapy following MDT and informed consent. While​ autoimmune diseases are not absolute contraindications to ICIs, meta-analyses demonstrate a 2–3-fold increased risk of severe immune-related adverse events (irAEs)—including disease flare and pneumonitis—in this population (17, 18). This risk, compounded by the patient’s heavily pretreated advanced disease and the paucity of evidence specific to Sjögren’s syndrome with gastric adenocarcinoma, supported a conservative approach.

The patient presented with fatigue and blurred vision starting in August 2023. Based on imaging signs and blood test results, a presumptive diagnosis of pineal metastasis, along with hypopituitarism, was established. The imaging characteristics of the pineal lesion, including its T1 isointensity and marked enhancement without calcification or cystic change, aligned closely with typical radiographic features of pineal metastases described in the literature (19–21). Additionally, BRAF mutation is a common driver mutation in gastrointestinal tract cancers but is exceedingly rare in primary pineal tumors (e.g., germinomas, pineocytomas). Its detection in cerebrospinal fluid (CSF) therefore supports gastrointestinal dissemination (22, 23). In the absence of histopathological confirmation, the pineal lesion is presumptively classified as a metastasis from gastrointestinal tract cancer, based on the patient’s history of gastric adenocarcinoma, concordant BRAF V600E mutational status, and characteristic imaging features. Pineal region tumors constitute approximately 3–11% of pediatric brain neoplasms and fewer than 1% of adult brain neoplasms (24–26). Metastatic cancer spreading to the pineal gland typically originates from lung cancer (27). There is a certain incidence from pancreas, esophagus, and bladder (19, 22, 23). Gastric adenocarcinoma rarely metastasizes to the central nervous system (CNS), with pineal metastasis being an extremely rare subtype. The pineal region interfaces with hypothalamic-pituitary regulatory centers. Significant fatigue suggested differentiation from routine treatment toxicity. Hormonal testing confirmed severe hypopituitarism, hypothyroidism, and adrenal insufficiency, supporting radiological suspicion of pineal metastasis. Without histopathological confirmation, the temporal link between pineal lesion progression and endocrine dysfunction, along with symptomatic improvement after targeted therapy and radiotherapy, indicates a likely role of mass effect on neuroendocrine structures or melatonin signaling disruption affecting the hypothalamic–pituitary–adrenal (HPA) and hypothalamic–pituitary–thyroid (HPT)​ axes (21, 28). However, a direct causal link is unproven, and alternative explanations like treatment-related endocrine toxicity or autoimmune dysregulation remain possible. Treatment options for pineal metastasis are limited. CNS-penetrant regimens and intrathecal therapy were avoided due to end-stage disease and poor tolerance. The patient instead received CyberKnife radiosurgery in September 2023, with concurrent systemic targeted therapy.

Serial biopsies and molecular profiling showed marked HER2 and CPS heterogeneity between primary and metastatic lesions, consistent with clonal evolution under therapeutic pressure (29, 30). Tissue-based analysis was limited to cutaneous and primary specimens, while the pineal lesion was profiled via CSF ctDNA, confirming BRAF V600E status; however, HER2 and CPS assessment remained unfeasible due to reliance on immunohistochemistry, highlighting the utility of liquid biopsy for inaccessible sites. Molecular data for hepatic and intra-abdominal lesions were unavailable, explaining the “Unknown” status in **Table 1**. HER2 heterogeneity may result from copy number variations or promoter methylation, influenced by therapeutic stress and the tumor microenvironment (31), while CPS discordance reflects regional variation in tumor-infiltrating lymphocytes (32). These findings emphasize that metastatic lesions are evolving and support repeated molecular profiling for adaptive treatment (33).

Fourth-line therapy was guided by serial ctDNA MRD monitoring, revealing a rising BRAF V600E VAF (0.5% to 13.2%), with no prior BRAF inhibitor exposure and refusal of chemotherapy. Dabrafenib plus trametinib, a validated regimen for BRAF V600E-mutant solid tumors (34, 35), was initiated after comprehensive counseling, achieving brief disease control (1.5 months). Given this limited efficacy, fifth-line therapy shifted to Disitamab vedotin-based treatment targeting HER2-low subclones (IHC 2+/FISH-non-amplified) identified in peripheral metastases. Disitamab vedotin, a HER2-targeted antibody-drug conjugate (ADC) conjugated with monomethyl auristatin E (MMAE) and to which the patient had no prior microtubule inhibitor cross-resistance, was combined with apatinib and S-1, yielding a partial response lasting 8 months (36). Sixth-line therapy substituted TAS-102 for S-1, resulting in stable disease for 4.5 months. With standard options exhausted, seventh-line therapy was driven by marked VAF escalation in both blood (25.66%) and CSF (47.67%), confirming a dominant BRAF-mutant pineal subclone. The regimen combined dabrafenib, Cetuximab, and anlotinib with concurrent CyberKnife radiosurgery. Overall, anti-BRAF therapy showed modest efficacy, raising the question of whether earlier integration of anti-HER2 therapy might have improved outcomes.

## Patient perspective

Upon noticing the initial skin lesions on her chest wall, the patient experienced significant anxiety, fearing disease progression and potential disfigurement. The subsequent onset of blurred vision and severe fatigue further exacerbated her distress, as she initially attributed these symptoms to the side effects of long-term chemotherapy rather than a new metastatic site. After being informed about the pineal gland metastasis and the complex endocrine dysfunction, including polyuria and adrenal insufficiency, she expressed concern regarding the prognosis but remained determined to continue treatment. Despite experiencing multiple lines of therapy and the inconvenience of hormone replacement, she reported that the improvement in her vision and the stabilization of her urine output greatly improved her quality of life. She actively participated in the decision-making process regarding the targeted therapy and expressed satisfaction with the multidisciplinary team’s efforts to manage her rare condition.

## Implications for clinical care

This case highlights that synchronous cutaneous and pineal metastases from gastric adenocarcinoma, although extremely rare, should be considered in patients with progressive disease and atypical neurological or dermatological symptoms. Timely imaging and molecular detection are crucial for early diagnosis. The marked molecular heterogeneity between primary and metastatic lesions supports the necessity of repeat biopsy and dynamic genotyping to guide personalized treatment. For heavily pretreated patients with multiple metastases, targeted therapy combined with local radiotherapy may help prolong survival and maintain quality of life. Endocrine disorders coincident with pineal metastasis require early identification and standardized hormone replacement therapy to avoid life-threatening complications.

## Limitations

This single-case report limits statistical generalizability, and outcomes cannot be definitively attributed to any single agent given the multimodal treatment sequence. Histopathological confirmation of the pineal lesion was precluded by high procedural risk, rendering the diagnosis presumptive despite molecular concordance in CSF. The patient’s longstanding Sjögren’s syndrome and extensive prior cytotoxic therapy represent additional confounders that further limit causal inference. Inferences regarding treatment sequencing remain speculative and hypothesis-generating.

## Conclusion

The concurrent presence of cutaneous and pineal metastases in gastric adenocarcinoma represents a rare clinical scenario, which is characterized by substantial molecular heterogeneity among the different metastatic lesions. Although CMs have demonstrated increasing responsiveness to systemic therapies such as immunotherapy, pineal metastases remain particularly challenging due to their deep-seated location and the paucity of effective treatment options. This case illustrates​ the potential value of comprehensive molecular profiling and multidisciplinary collaboration in guiding individualized therapeutic decisions for patients with heavily pretreated metastatic gastric cancer. Future research should prioritize the elucidation of the mechanisms underlying metastatic tropism and developing targeted therapies for rare metastatic sites, including the pineal gland.

## Data Availability

The raw sequencing data generated in this study have been deposited in the Genome Sequence Archive for Human (GSA-Human) database under accession number HRA017759.
