# Supplementary material for: Case Report: Synchronous cutaneous and pineal metastases from gastric adenocarcinoma—inter-lesion biomarker heterogeneity and multi-line targeted therapy
Source: Front Oncol. 2026 Aug 12;16:1924850. doi: 10.3389/fonc.2026.1924850 (PMC13506412; doi:10.3389/fonc.2026.1924850)
Supplement: Supplementary file 2 [file Table1.doc]

**Additional file 3: Tables S1–S3**

**Table S1. Diagnosis of adrenal cortical insufficiency.**

| **Inspection Number** | **1019307552** | **1025131285** | **1031385893** | **1031499215** | **1031682906** |
| --- | --- | --- | --- | --- | --- |
| Reception Date | 2022-05-11 | 2022-10-13 | 2023-08-27 | 2023-09-03 | 2023-09-13 |
| Plasma Cortisol Determination (8:00) | 304.50 | 275.20 | 41.58 | 186.30 | 608.90 |
| Plasma Adrenocorticotropic Hormone Determination (8:00) | 17.19 | 15.13 | 8.59 | 9.10 | 3.30 |

| **Inspection Number** | **1019307552** | **1025131285** | **1031385893** | **1031499215** |
| --- | --- | --- | --- | --- |
| Reception Date | 2023-08-28 | 2023-08-30 | 2023-09-06 | 2023-09-13 |
| Plasma Cortisol Determination (15:00) | 36.45 | 1260.00 | 502.90 | 537.60 |
| Plasma Adrenocorticotropic Hormone Determination (15:00) | 1.89 | 1.95 | <1.00 | 2.00 |

Plasma cortisol (nmol/L) and plasma adrenocorticotropic hormone (pg/mL) levels measured at 08:00 and 15:00 during follow-up. Hydrocortisone was administered by intravenous infusion at a dose of 100 mg. After two days, the dose was adjusted to 50 mg for intravenous infusion. Subsequently, it was gradually switched to oral administration with 40 mg in the morning and 20 mg in the evening. Eventually, the maintenance dose was set at 10 mg in the morning and 5 mg in the evening.

**Table S2. Hypothyroidism treatment with levothyroxine tablet replacement therapy**

| **Test Number** | **1019307452** | **1025131284** | **1031447428** | **1031499214** | **1031903163** |
| --- | --- | --- | --- | --- | --- |
| Reception Date | 2022-05-10 | 2022-10-13 | 2023-08-30 | 2023-09-03 | 2023-09-26 |
| Serum Thyroglobulin Antibody Assay | <15.0000 | <15.0000 | 0.9000 | 1.0800 | 0.0000 |
| Serum Thyroid Peroxidase Antibody Assay | 47.2000 | 31.7000 | 36.0000 | 1.0000 | 53.0000 |
| Serum Thyroid Stimulating Hormone Assay | 2.4110 | 4.1960 | 0.1070 | 0.1289 | 0.0160 |
| Serum Free Thyroxine Assay | 15.1400 | 14.1100 | 7.4300 | 7.2900 | 11.5700 |
| Serum Free Triiodothyronine Assay | 4.6000 | 4.6700 | 2.3200 | 2.8300 | 3.3300 |

Serum thyroglobulin antibody (IU/mL), serum thyroid peroxidase antibody (IU/mL), serum thyroid stimulating hormone (mIU/L), serum free thyroxine (pmol/L), and serum free triiodothyronine (pmol/L) levels monitored during levothyroxine dose adjustment.

**Table S3. Changes in urine osmolality.**

Urine osmolality (mOsm/kg H₂O) measured by the freezing point depression method, before and after desmopressin administration. The patient presented with polyuria, with a 24-hour urine volume ranging from 6000 to 8000 milliliters, accompanied by an intense sense of polydipsia. The daily fluid intake was approximately 6000 milliliters. Notably, the levels of blood ions and blood pressure remained essentially normal. Based on consultation with staff from the endocrinology department, it was recommended to administer ⅛-tablet of desmopressin. Following this treatment, the daily urine volume was successfully maintained within the range of 2000 to 3000 milliliters.

| **Test Number** | **1031445656** | **1031499210** | **1031903160** |
| --- | --- | --- | --- |
| Reception Date | 2023-08-30 | 2023-09-03 | 2023-09-26 |
| Urine Osmolality | 67 | 455 | 350 |
